# Deep-Learning and Vibration-Based System for Wear Size Estimation of Railway Switches and Crossings

**DOI:** 10.3390/s21155217

**Published:** 2021-07-31

**Authors:** Taoufik Najeh, Jan Lundberg, Abdelfateh Kerrouche

**Affiliations:** 1 Department of Civil, Environmental and Natural Resources Engineering, Luleå University of Technology, SE-971 87 Luleå, Sweden; jan.lundberg@ltu.se; 2 School of Engineering and the Built Environment, Edinburgh Napier University, 10 Colinton Road, Edinburgh EH10 5DT, UK; a.kerrouche@napier.ac.uk

**Keywords:** switches and crossings, wear measurement, deep learning, LSTM, ResNet vibration sensors

## Abstract

The switch and crossing (S&C) is one of the most important parts of the railway infrastructure network due to its significant influence on traffic delays and maintenance costs. Two central questions were investigated in this paper: (I) the first question is related to the feasibility of exploring the vibration data for wear size estimation of railway S&C and (II) the second one is how to take advantage of the Artificial Intelligence (AI)-based framework to design an effective early-warning system at early stage of S&C wear development. The aim of the study was to predict the amount of wear in the entire S&C, using medium-range accelerometer sensors. Vibration data were collected, processed, and used for developing accurate data-driven models. Within this study, AI-based methods and signal-processing techniques were applied and tested in a full-scale S&C test rig at Lulea University of Technology to investigate the effectiveness of the proposed method. A real-scale railway wagon bogie was used to study different relevant types of wear on the switchblades, support rail, middle rail, and crossing part. All the sensors were housed inside the point machine as an optimal location for protection of the data acquisition system from harsh weather conditions such as ice and snow and from the ballast. The vibration data resulting from the measurements were used to feed two different deep-learning architectures, to make it possible to achieve an acceptable correlation between the measured vibration data and the actual amount of wear. The first model is based on the ResNet architecture where the input data are converted to spectrograms. The second model was based on a long short-term memory (LSTM) architecture. The proposed model was tested in terms of its accuracy in wear severity classification. The results show that this machine learning method accurately estimates the amount of wear in different locations in the S&C.

## 1. Introduction

Railway tracks represent a complex piece of infrastructure and are installed to last for a long time. Once they are put in place, it is very problematic and costly to maintain them at a high standard. It is very important to monitor any urgent maintenance of the Switch and Crossing (S&C), as insufficient maintenance may present the main source of track irregularity, which can not only affect passenger comfort [1], but also deteriorate the vehicle–infrastructure interaction performance [2]. Therefore, the return on investment (ROI) in a manageable timeframe of such systems is extremely dependent on the maintenance strategy and decisions. The aim of the study presented herein was to enhance the existing knowledge of railway wear monitoring by providing novel techniques to monitor the surface deterioration and damage induced by wear effects.

In the railway wear monitoring literature [3,4,5,6], two main categories of monitoring approaches can be found, namely direct and indirect approaches. The direct approaches are mainly based on the use of digital photography to monitor specific locations on the rail [7]. Images are captured using optical devices such as high-speed cameras, after which conventional image processing tools and algorithms are applied to extract directly the information required concerning deterioration caused by the wear process [8,9]. On the other hand, the indirect approaches estimate the wear by implementing a data acquisition system to collect signals that can be generated from the dynamic wheel–rail interaction during operation. The most frequently collected signals are vibration [10], force [11], and speed signals. The measured signals are then used as input fed into a mathematical wear model or signal-processing tools to extract relevant features to estimate the amount of wear [12].

From a mechanical point of view, there is a direct cause–effect relation between wear and rail corrugation [13]. Originally, rail roughness has a large distribution of wavelengths with small amplitudes. However, because of the periodic wear caused by the wheel–rail interaction along the longitudinal direction, a dynamic change in the roughness will accrue through periodic waviness and irregularities with high amplitudes [14], resulting in what is called rail corrugation. 

In the present study, rail corrugation was used as the key feature for wear assessment. Corrugation is generally caused by the mechanism of differential wear, where the corrugation troughs are more exposed to wear than the peaks. In most cases, rail corrugations have quite a fixed wavelength produced by friction change and normal contact force fluctuations. Corrugation results in higher vibration signals and noise levels emanating from railway vehicles [15], and the vibration will be a source of information that can reflect the amount of rail wear.

In this study, special attention was paid to the optimal mounting location for the accelerometers. The aim of the study was to investigate the possibility of evaluating the amount of wear of the entire S&C indirectly using accelerometers embedded inside the point machine. The most common solutions used for S&C wear measurement are systems located onboard the car-body and installed on the bogie [16], and wayside systems using a network of sensors distributed along the S&C track [17]. Comparing these two types of systems [3], it has been found that wayside sensors are overwhelmingly more effective than onboard monitoring systems. Furthermore, placing accelerometers or other types of sensors as an integrated component of the point machine has the advantage that they are therefore perfectly protected from harsh weather conditions such as snow and ice, as well as damage caused by service vehicles during the operation process. Another benefit is that the collected vibration signals can be used both to detect damage and degradation related to the railway track and defects in the point machine components. An additional advantage is related to the complexity of installing the data acquisition system; by placing the sensors in this optimal location, less wiring and packaging are needed. Furthermore, the electrical power supply can be easily driven from the point machine itself. 

In 2005, a dissertation was published [18], written by Argo Rosin, which deals with control and steering as well as operational diagnostics for light electric transports by rail. However, the dissertation only concerns reliability analyses from measuring systems mounted on locomotives and wagons. Mathematical models have been developed in the dissertation that analyze input data from sensors on locomotives and data have also been successfully transmitted to fixed analysis stations. However, the important problem of the wear is not addressed, and the S&C investigation is very superficial. The main drawback of this onboard approaches is the noise level originated from the bogie structure. In 2017, a dissertation was published in England [19], written by Marius Florin Rusu, which deals with automatic inspection approaches of S&Cs. The dissertation provided a good motivation for the needs of new measurement methods and a theoretical analysis about the optimal location of the sensors but without any real case study. The dissertation claims that the point machine is theoretically a suitable location to install the sensors with a view to forecast the state of the S&C.

However, the major disadvantage of having the sensors housed inside the point machine is that the sensors are, to a varying extent, distant from the actual location of the defects. As a result of this, the collected signal can be noisy and not strong enough to convey useful information about the wear evolution near the crossing part of the S&C, for example. Optimally, one would need to install the accelerometers as close as possible to the expected locations of defects and in several positions along the S&C track. However, installing a wear monitoring system of this type that would cover the entire Swedish railway network with its more than 14,000 S&Cs would not be possible because of the unrealistic installation costs [20,21,22]. Consequently, installing a monitoring system consisting of sensors as an embedded device that can be delivered with the point machine seems to be a more feasible solution.

Artificial intelligence (AI) and more specifically deep-learning algorithms have to a high degree captivated the interest of academics and companies in almost all fields. These algorithms, with their diversity of architectures and concepts, can model many real-world problems if they are provided with relevant and well-structured data [23]. Furthermore, AI is now a key feature of many scientific achievements in railway track condition monitoring. Hitoshi [16] developed an onboard sensing device for fault classification using the support vector machine (SVM) method. Hamid et al. [24] proposed an artificial neural network methodology to predict the track geometry degradation.

## 2. Deep-Learning Algorithms for Feature Learning

### 2.1. Long Short-Term Memory (LSTM) for Feature Learning

**Figure 1:**
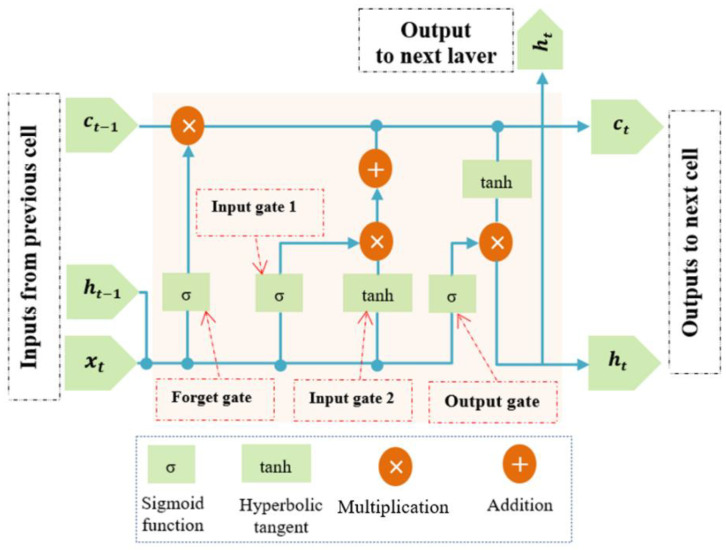
Conventional design of LSTM cell.

The present study made use of the well-known deep-learning architecture called long short-term memory (LSTM), which belongs to the family of recurrent neural networks (RNNs). As has been illustrated in the literature [18], RNN models are good at reducing frequency variations. LSTM is a distinct version of RNN which deals with the vanishing gradient problem, which considers the time notion, and which solves the problem of storing short-term data over long periods of time. The LSTM architecture is more appropriate for the temporal modelling of sequence data [25]. The main idea behind the LSTM concept is the memory block that memorizes its state over the training process. With the memory block introduced, it is possible to keep old features gained at the beginning of the training phase and fresh features collected by the end of the training. The workflow of one LSTM cell is plotted in Figure 1 and it can be mathematically formulated as follows:

(1) $$c_{t}=f_{t}⊗c_{t-1}+i_{t}⊗{\tilde{c}}_{t}$$

(2) $$h_{t}=o_{t}⊗\tanh\left(c_{t}\right)$$

(3) $$\mathrm{where}\;f_{t}=\sigma \left(W_{fh}h^{t-1}+W_{fx}x^{t}+b_{f}\right)$$

(4) $$i_{t}=\sigma \left(W_{ih}h^{t-1}+W_{ix}x^{t}+b_{i}\right)$$

(5) $${\tilde{c}}_{t}=\tanh\left(W_{ch}h^{t-1}+W_{cx}x^{t}+b_{c}\right)$$

(6) $$o_{t}=\sigma \left(W_{oh}h^{t-1}+W_{ox}x^{t}+b_{o}\right)$$

$W_{fh}$, $W_{fx}$, $W_{ih}$,$\;W_{ix}$, $W_{ch}$, $W_{cx}$,$\;W_{oh}$ and $W_{ox}$ are the weights for the forget gate, input gate, input modulation, and output gate, respectively. The cell has three inputs made available from the previous propagation. The input is processed with “σ” and “tanh” internal gates, which are ruled by the hyperbolic tangent function and the sigmoid function, respectively. *b_f_*, *b_i_*, *b_o_*, and *b_c_* are the biases matrices and they are not time-dependent, this implies that these matrices do not update from one-time step to another.

Figure 1 shows the input and outputs flow of an LSTM for one timestep. This is a single timestep input, output ruled by Equations (1) and (2). Each LSTM cell has an input *x_t_*, *h_t−_*_1_, and *c_t−_*_1_ are the inputs from the previous timestep LSTM. *o_t_* illustrate the output of the LSTM cell for the current timestep. The LSTM also produces the *c_t_* and *h_t_* for the feeding of the next time step LSTM. Based on the present input *x*, the internal state c and the hidden state *h*, the internal gates will decide as to the amount of information that can be updated into the hidden state h and the cell state *c*. This behavior grants the LSTM cell the ability to uncover new key features and remove irrelevant information.

### 2.2. The Residual Neural Network (ResNet) for Feature Learning

**Figure 2:**
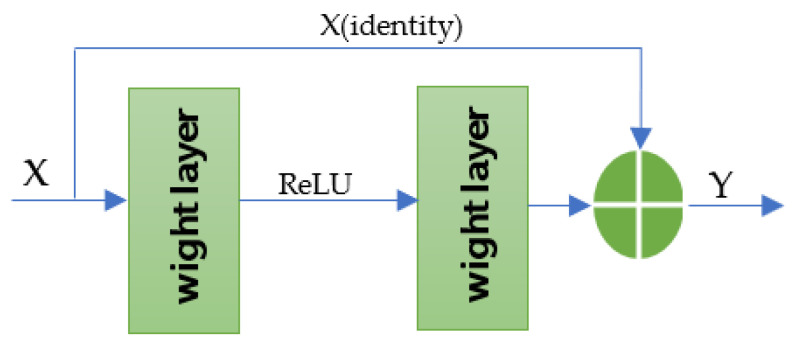
Residual block in a ResNet architecture model.

The residual neural network (ResNet) is one of the most successful deep networks and belongs to the family of convolutional neural networks (CNNs). The first draft of the ResNet was proposed in [26]. The main basis for its architecture is the residual learning which is the use of skip connections, also called identity skip function, to jump over some layers. The residual concept adds an explicit identity connection throughout the network to help the network learn the required identity mappings as shown in Figure 2. Adopting this approach, the network will be more dynamic and can decide how deep it needs to be to reach the highest accuracy. Even though this new concept will introduce a new parameter to the network, it will not add any computational problem. Moreover, because it has a deeper layer’s presentation, the ResNet makes it possible to design deeper learning applications that deal with more complicated real-world problems. Furthermore, it has been shown in the literature that this type of deep network facilitates faster convergence than that achieved by a CNN which does not have a skip connection function [27,28,29].

## 3. Materials and Methods

### 3.1. Experimental Set Up and Sensors Placement

**Figure 3:**
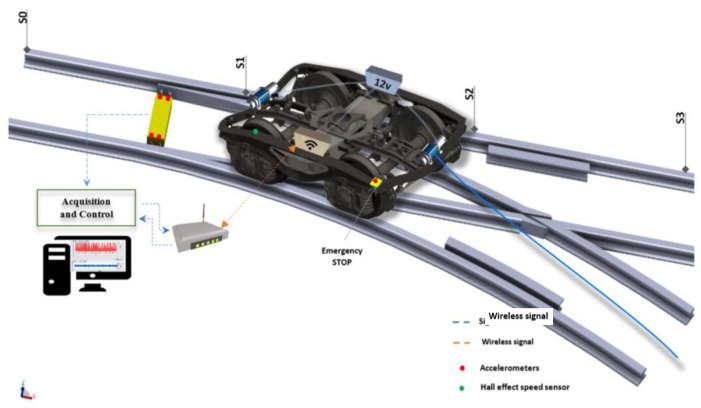
Principle sketch of the test switch (bogie picture: courtesy of Igor Antolovic at Kockums Industrier AB).

The basic idea behind this study is that the movements of trains are affected by the degradation of the S&C. This degradation can result in defects occurring during normal railway operations. The resulting vibrations happen as a dynamic response to the wheel–rail interaction. If the rail or the wheel profile changes over time because of regular degradation, then the vibration response will also change. This means that the trains’ vibrations can be correlated with the health condition of the S&C. Consequently, measuring the vibrations will lead to an estimation of the amount of wear. In this study, six accelerometers were used to acquire the vibration signals. It has been reported in the existing literature [19] that the normal range of frequencies of railway infrastructure is quite low and hardly exceeds 10 kHz. However, to study high frequencies further, two accelerometers with the higher frequency of 37 kHz were used. A principle sketch of the test rig with its point machine used in the present study is provided in Figure 3.

Figure 3 shows the 6-tonne bogie used as part of the test rig to perform the measurements. This two-axle bogie (with an axle distance = 2.5 m) can move along the turnout in the diverging and the through directions using two electrical winches installed at the front and at the back of the bogie structure, respectively. Using a metallic cable hooked into the sleeper, the bogie can be moved in the desired direction (Figure 3). The recorded speed was between 0.016 m/s and 0.018 m/s. To drive the winches, a petrol-powered electric generator mounted on the bogie is used. The entire S&C path is divided into three sections: S0S1, S1S2, and S2S3, as shown in Figure 3. S0S1 includes the point machine, S1S2 includes the middle section, and S2S3 includes the crossing part, and the lengths of these three sections are 13.85 m, 10.14 m and 11.40 m, respectively.

**Figure 4:**
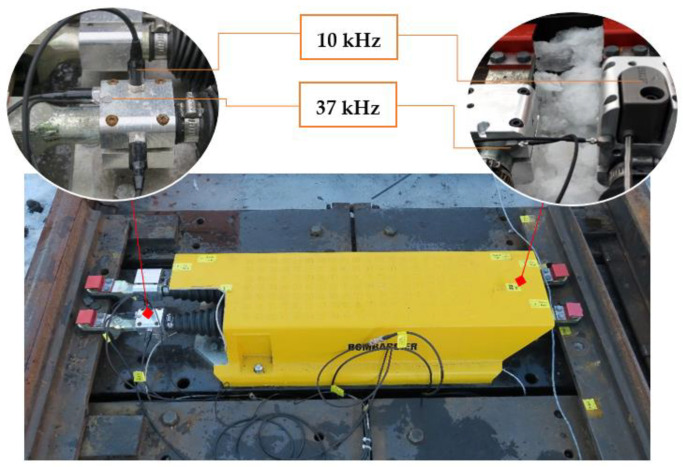
Point machine with the installed accelerometers.

In this study, several accelerometers were used, and the locations of the sensors on the S&C structure were based on a compromise between an optimal placement based on the probable vibration directions and the realistic placement possibilities available (see Figure 4).

Figure 4 shows the installation of all the accelerometers with the acquisition unit as an extra integrated component for the point machine used with the test rig. The sensors were distributed in a such way as to keep a straight contact with the rest of the S&C elements to guarantee faultless acquisition of the vibration data. The sensors were mounted using customized supports considering the desired direction of the accelerometer. As shown in Figure 4, the accelerometers were mounted in such a way as to detect the longitudinal and vertical vibration and they are balanced on the left-hand and the right-hand sides of the different rods of the point machine. 

### 3.2. Data Aquisition for Vibration and Speed

**Figure 5:**
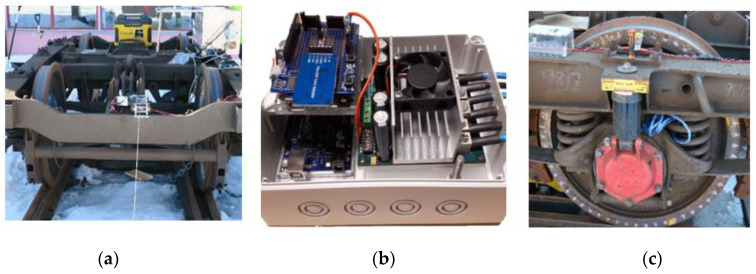
(**a**) Bogie with two winches and an electrical power supply, (**b**) remote control unit, and (**c**) tachometer.

The speed was measured by a customized tachometer using a Hall effect sensor (A3144), several neodymium magnets (20 mm × 10 mm × 1.5 mm thick), and an Arduino Uno with wireless communication capability (Figure 5). Every second, the revolutions per minute (RPM) of the front wheel were measured and the readings were sent wirelessly to the main computer to be synchronized with the vibration data acquisition. Another Arduino Uno (WiFi Rev2, Kjell & Company Elektronik AB, Malmo, Sweden) was used with a programmable Sabretooth motor controller (2X60A) to control remotely the two DC motors driving the winches used to move the bogie forward and backward. To ensure that the vibration data acquisition and speed data acquisition were synchronized with the same timestamp, the measurements were triggered at the same time as the motor was powered.

**Figure 6:**
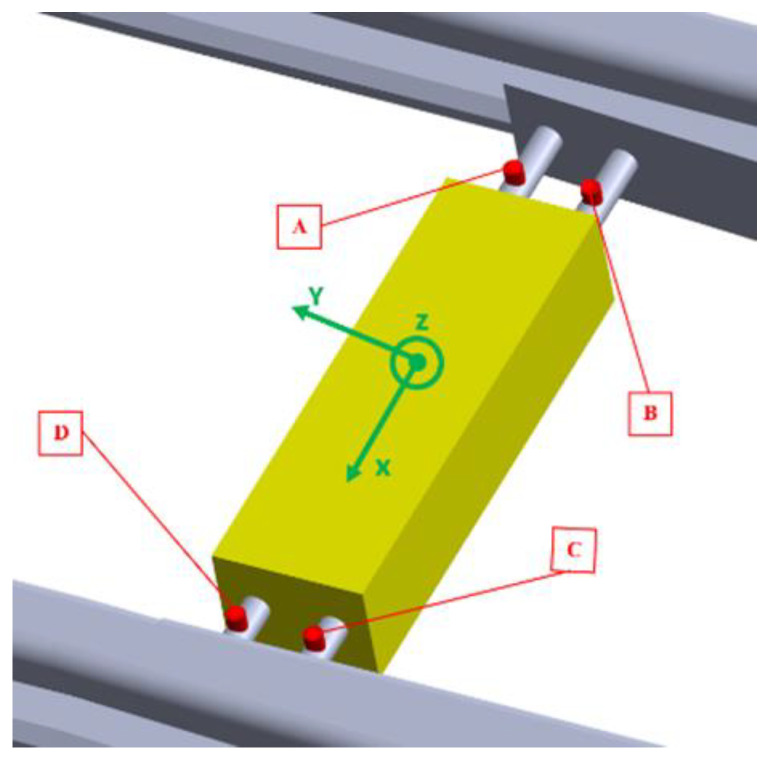
Locations (**A**–**D**) and directions of the accelerometers.

The vibration data were then collected by a National Instruments data acquisition unit connected to accelerometers. The test setup for the test rig is presented in Figure 3. The National Instruments platform (a Compact DAQ 9174) was used for vibration data acquisition, and two modules were required to connect the accelerometers. The experiment was carried out with different class of piezoelectrical accelerometers connected to a Compact DAQ 9174 (National Instruments Sweden AB, Stockholm) and providing vibration signal acquisition with a sampling rate of up to 51 kHz for each channel. To create a good surface-to-surface contact between the accelerometers and the point machine’s rod, an adapted support was made which had much the same shape and size as the different accelerometers. Figure 6 depicts the locations (A, B, C, and D) and the directions of the accelerometers installed on the point machine, see Table 1 for additional properties.

### 3.3. Wear Severity Classification Using LSTM and RESNET

**Figure 7:**
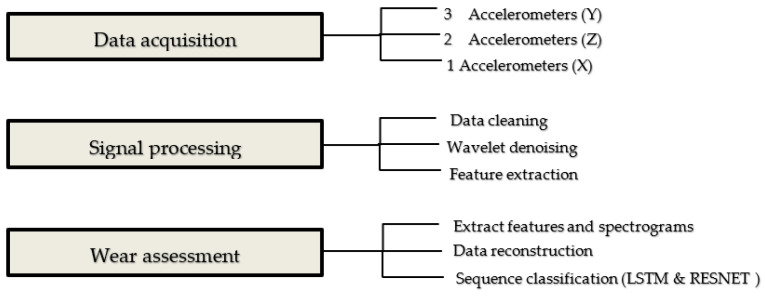
The algorithmic workflow of the proposed approach.

The aim of this research was to develop a strategy for measuring the severity of the wear on the rail indirectly. The method for detecting wear includes vibration data acquisition, pre-processing of the vibration signals and then assessment of the wear severity. Figure 7 presents the algorithmic workflow of the approach, and the principal steps are described below.

The sampling rate used for the vibration data was 51.2 kHz and that used for the speed data was 1 Hz. The data contained vibration measurements in three directions using accelerometers installed on the point machine in several locations. The speed recording was synchronized with the vibration recording, and the final measurement output was a data file with six columns for the vibration input channels, one column for the time stamp, and one column for the speed value. Wavelet denoising or wavelet thresholding was applied to the vibration signals using the MATLAB Signal Toolbox. The essential idea behind the denoising algorithm is the decimated wavelet transform. Wavelet denoising pinpoints features in the vibration data to different scales, which then makes it possible to keep important signal features while removing the noise. The basic idea of this method is to construct a new presentation of the original signal using predefined signals to concentrate the signal to obtain the wavelet coefficients. Then it is possible to localize the small values’ coefficients and remove them to reduce the noise in the signal without affecting the main features carried by the measured signal. To reconstruct the final signal with remarkable noise reduction, we used the inverse wavelet transform. 

Two approaches will be used in this section, the first approach is based on several features extracted from the vibration data and then the LSTM network will be fed by these features as a tabular data. The second approach is based on the spectrogram images as an input to train the ResNet model. Both proposed algorithms are tested with the data set to identify which one is more suitable and more efficient in terms of wear severity assessment accuracy.

#### 3.3.1. LSTM Model for Wear Severity Classification

**Figure 8:**
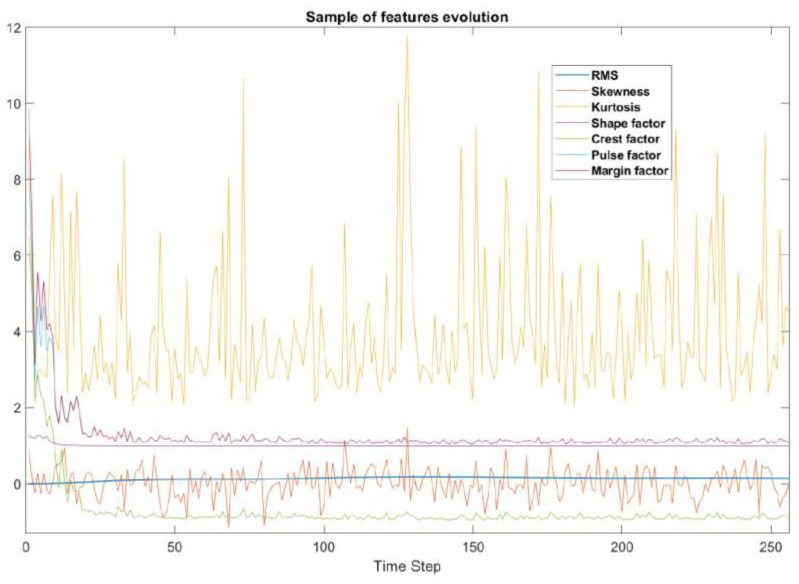
A sample of the feature evolution for one sample block of data.

To make the data ready to be fed into a deep-learning model, the raw data were divided into windows of three seconds. As the run lasted for 120 s, we then had 40 windows of 153,600 timestamps. Each window was reshaped into 256 blocks of 600 values each and the windows were used to extract several features commonly used for defect detection in the bearing fault diagnosis field [20]. The result was a 3-dimensional matrix (windows, time steps, and features). All in all, we selected seven-time domain features. Figure 8 shows a sample of the feature evolution for one sample block of data over 256-time steps. The corresponding names and formulas for these features are listed in Table 2.

As an example, all results are presented in Figure 8, the purpose here is to give an overview about the typical behavior of the selected features, it is shown that most of the features are relevant to vibration signal which reflect the condition of the S&C itself.

The extracted features will be used to feed the LSTM model, in this study the model was designed with MATLAB 2020b using the Deep-Learning Toolbox (MathWorks, Massachusetts, USA) with the support of a computer whose CPU had 4 gigabits of memory. The first step involved splitting the data, allocating 60% of the data for learning and 40% for testing, with the load of the training data containing 208 sequences of dimension 7 of a fixed length equal to 265. Another categorical vector of labels was introduced corresponding to the four wear levels. The learning time for the model was about 12 min and the inference time using a sequence of vibration data was about six seconds. For the assessment of the model performance, the following formulation was employed:

(7) $$Accuracy=\frac{\sum (Level_{pred}==Level_{test})}{Number\;of\;test\;sequences}$$

#### 3.3.2. ResNet Model for Wear Severity Classification

Our intention in using a ResNet model was to solve the same problem using another CNN architecture. The main difference between the first approach and the new one is the way in which the data are fed into the machine learning model and the way in which the data are pre-processed.

**Figure 9:**
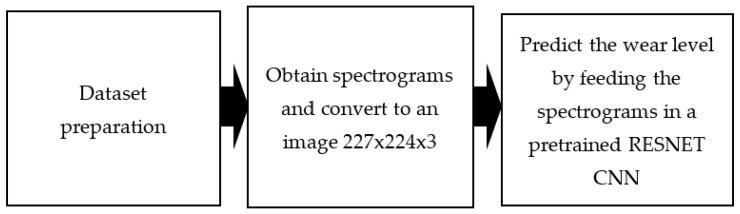
Workflow of the ResNet-based model.

In the previous method, the vibration signals are used as tabular time series data to be fed into the LSTM model after structuring and extracting the needed features from the raw signals. For the ResNet model, the raw data are converted into spectrograms, which are then used as input for training the model. We have selected the ResNet model because it handles images better. The workflow of the second proposed approach is shown in Figure 9. In the ResNet model the same method is applied for processing the data as was applied in the previous approach with the LSTM model, but the vibration data are changed to images instead of tabular time series data. 

## 4. Results and Discussion

### 4.1. Vibration Measurements

To protect the installation and the cables shown in Figure 6, all the accelerometers were located inside the point machine housing. A single-board computer (SBC) was used with a LabView graphical user interface (GUI) to transfer commands and save the vibration data. Various measurements were performed (Table 3) while the bogie was dragged with the electric winch mounted on the bogie structure. The data were recorded as the bogie moved over the middle part of the S&C in the turnout direction. The same scenario was repeated for four different degrees of wear.

**Figure 10:**
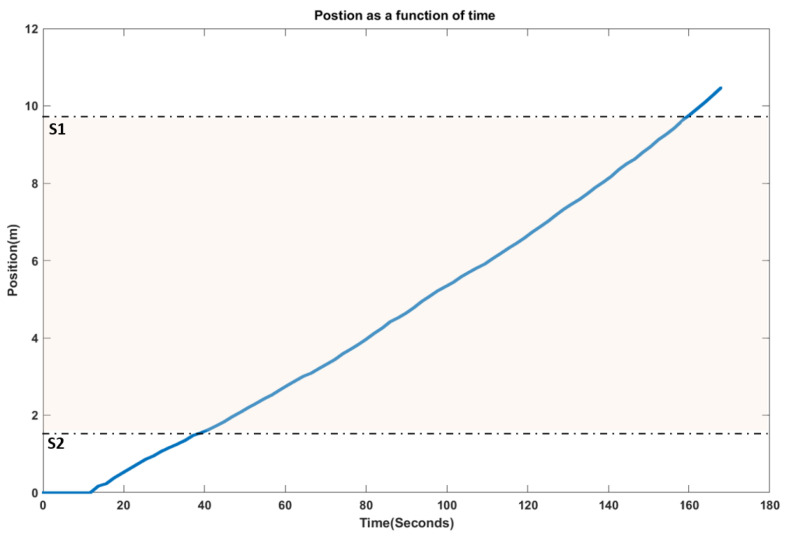
Position of the bogie over time.

The vibrations resulting from the wheel–rail interaction, as well as the speed, were collected at the point machine and saved on the hard drive. The speed was used to find accurately the exact location of the bogie from the start to the end of the test (Figure 10). Then it was possible to correlate the vibration response with the defect location on the rail. We only used the data recorded when the bogie passed over the middle section (S1S2 = 8 m), where the artificial wear had been created.

### 4.2. Wear Measurement

**Figure 11:**
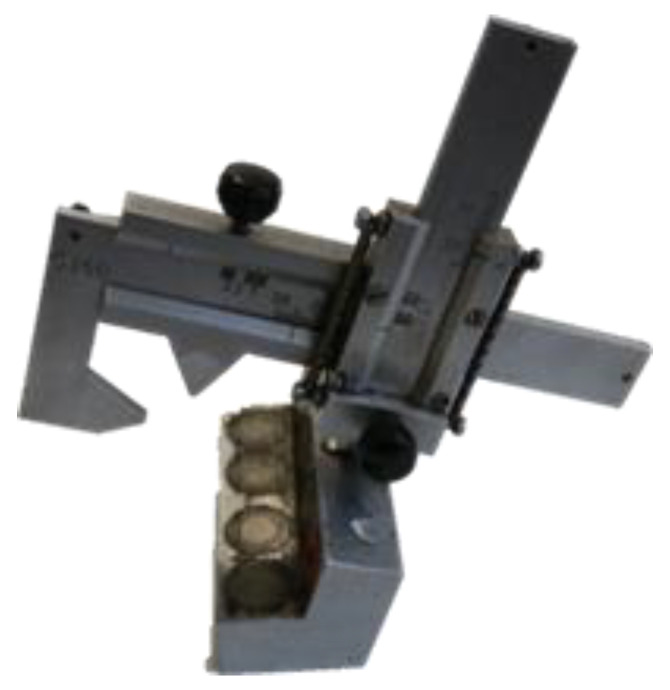
SJ50: the tool used to measure the rail wear.

The actual amount of wear in the S&C was measured with a special type of mechanical measuring tool used by a Swedish railway maintenance contractor. The tool (an SJ50) was placed using magnets on the top of the railhead in the location where the artificial wear had been created. The wear measurements were performed in several locations in both the vertical and the horizontal directions (see Figure 11). Table 4 shows the average evolution of the artificial wear for all the parameters on the entire S&C. X0, X3, X6 and Z0 are specific settings to take the measurement at one location on the rail.

### 4.3. Wear Measurement Using LSTM Model

**Figure 12:**
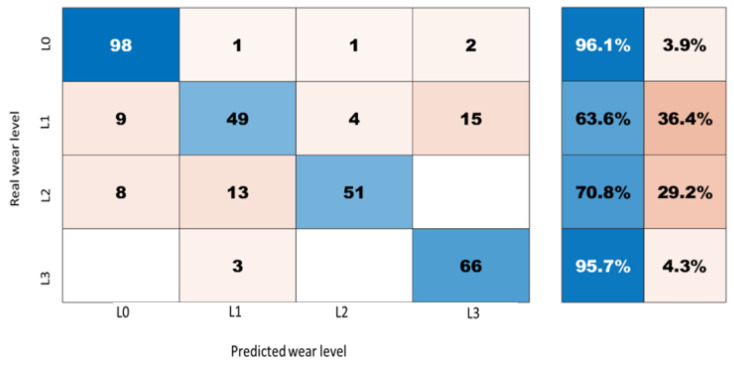
Confusion matrix for the wear classification using LSTM model.

As shown in the confusion matrix (Figure 12), the obtained LSTM model provides satisfactory results with an average accuracy for all the testing data of 88%. The classification is better when identifying the original wear level (L0) and the last level (L3), where we have an accuracy of 96.1% and 95.7%, respectively. However, for (L1) and (L2) the results indicate that there are some problems separating these low levels of wear (in the range of 1–2 mm) from each other. Surprisingly, using learning did not result in a satisfactory correlation between the wear and the extracted features. One explanation for this is the change in the vibration response after the large change of the wear size between the original state of the rail, level (L0) and level (L1). Furthermore, the last wear level is the case where we have removed the largest amount of material from the rail to create artificial wear, which is why the vibration response for this case is quite different from the response for the original level of wear. However, if this level of detail in the wear detection (1–2 mm) is required, more research is needed to explain this behavior.

In this paper, we did not apply any specific task for hyperparameters optimization. However, the main hyperparameters such as the number of training epochs and the size of training batches are merely tuned to interpret the accuracy of the results. A simple comparison shows that the choice of setting the number epochs and the size of training batches respectively to 100 and 32 is better than the tested alternatives.

The results obtained using the LSTM model show that extracting the time domain features from the signal, i.e., the RMS, kurtosis, skewness, crest factor, shape factor, impulse factor, and clearance factor, can be a good approach for indirectly quantifying the amount of wear on the railway track. There are other features which can be used, for instance frequency domain features, but if one intends to run the LSTM model locally on an edge computing and low computing capability, then it is better to keep the computation cost low.

### 4.4. Wear Measurement Using ResNet Model

**Figure 13:**
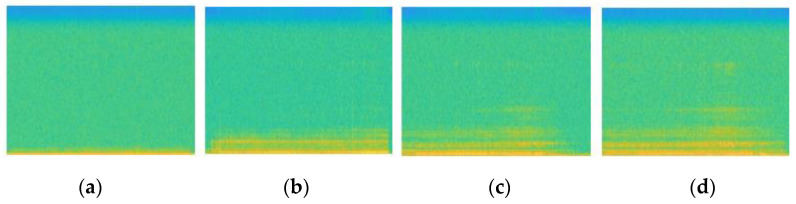
Spectrograms obtained from the vibration data for different level of wear: (**a**) no wear, (**b**) first level, (**c**) second level, (**d**) third level.

In this study, the convolutional neural network ResNet was used. The architecture of our ResNet model is 18 layers deep and was designed with MATLAB 2020b using the Deep-Learning Toolbox. We used a pretrained version of the ResNet18 which had been trained on more than a million images from the ImageNet database. The pretrained network had learned rich feature representations for a wide range of images. The input of the network had an image format. The vibration signal from each run was converted to a spectrogram, which was then saved as an image with a size of 224-by-224. All the images were labelled regarding the wear level in question (Figure 13) and divided into two datasets, one containing 60% of the data for learning and the other containing 40% of the data for testing, keeping the same configuration as for the LSTM model and the same hardware. For the assessment of the model performance, the same formula, Equation (7), was used.

**Figure 14:**
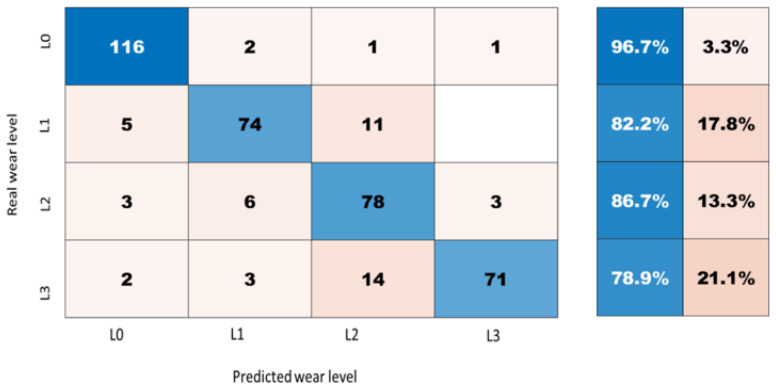
Confusion matrix for the wear classification using ResNet model.

The ResNet-18 takes the spectrograms as an input for the indirect extraction of features with respect to the wear level. The confusion matrix shows how accurate the ResNet is (Figure 14). Using this CNN architecture, the model exhibits slightly better accuracy than the LSTM model. In Figure 14, it can be observed that the ResNet can accurately distinguish the correct level of wear on the railway track among all the detailed levels of wear. On the other hand, the LSTM model fails to some extent, in that, for example, it does not succeed in differentiating between the levels L1/L0, L1/L3 and L2/L1. 

## 5. Conclusions

Switches and crossings (S&Cs) are a crucial part of the railway infrastructure and are subjected to various forms of wear which considerably decrease the useful operational life of the track. The first task in this study was to confirm the assumption that it was possible to measure vibrations that would reflect the amount of wear in the entire S&C. The second task was to investigate how the measurement accuracy would be affected by the distance between the sensor and the wear location. 

This study demonstrates that measuring the vibrations is an effective and promising way to monitor indirectly the amount of wear of the S&C, which is a critical component of the railway infrastructure. Compared with other existing approaches based on vibration measurement of several specific locations on the rail or onboard systems which are using sensors located on the wagons, the major disadvantages of these approaches are low reliability in harsh conditions, low accuracy, and high costs. Deep-learning applications based on the LSTM and ResNet neural network are proposed. 

The results show that the deep-learning solution can estimate the amount of wear in the middle section in the S&C with an acceptable accuracy. In future this solution will be developed and tested in a real S&C. The results will be used by a railway owner to implement an integrated platform within their systems, to predict the wear evolution and monitor and analyze the short-term and long-term condition of S&Cs.

## Figures and Tables

**Table 1:** Position, frequency range, and direction of the accelerometers.

Position	Accelerometer	Frequency (kHz)	Direction
C	KS91C1	37	Z
A	608A11	10	X
A	608A11	10	Y
A	608A11	10	Z
B	SKF 2310T	10	Y
D	SKF 2310T	10	Y

**Table 2:** Time domain features.

Features	Formula
Root Mean Square (RMS)	$X_{rms}=\sqrt{\frac{1}{N}\sum_{i=1}^{N}x_{i}{}^{2}}$
Skewness	$X_{skew}=\frac{\sum_{i=1}^{N}{\left(x_{i}-m\right)}^{3}}{\left(N-1\right)\sigma^{3}}$
Kurtosis	$X_{kurt}=\frac{\sum_{i=1}^{N}{\left(x_{i}-m\right)}^{4}}{\left(N-1\right)\sigma^{4}}$
Shape factor	$X_{shape}=\frac{\sqrt{\frac{1}{N}\sum_{i=1}^{N}x_{i}^{2}}}{\frac{1}{N}\sum_{i=1}^{N}\left|x_{i}\right|}$
Crest factor	$X_{crest}=\frac{max\left|x_{i}\right|}{\sqrt{\frac{1}{N}\sum_{i=1}^{N}x_{i}^{2}}}$
Impulse factor	$X_{impl}=\frac{max\left|x_{i}\right|}{\frac{1}{N}\sum_{i=1}^{N}|x_{i}|}$
Clearance (Margin) factor	$X_{clear}=\frac{max\left|x_{i}\right|}{{\left(\frac{1}{N}\sum_{i=1}^{N}\sqrt{\left|x_{i}\right|}\right)}^{2}}$

*m*: mean, σ: standard deviation.

**Table 3:** Measurement scenarios.

When	Repetition
Orig. wear level ^1^	4
1st wear level	3
2nd wear level	3
3rd wear level	3

^1^ The original condition of the S&C.

**Table 4:** Different levels of artificial wear.

Tool Position	Orig. Wear Level	1st Wear Level ^1^	2nd Wear Level ^1^	3rd Wear Level ^1^
X0	0.51	1.31	2.62	3.82
X3	1.63	2.36	4.48	5.92
X6	4.30	4.54	6.54	8.00
Z0	0.96	0.94	0.96	0.96

^1^ All measurements are in millimeters.

## Data Availability

Not applicable.
